# CRISPR–Cas9 Therapeutics in Early Clinical Development: Delivery and Molecular Diagnostics

**DOI:** 10.3390/cells15070644

**Published:** 2026-04-02

**Authors:** Adrianna Rutkowska, Tadeusz Strózik, Tomasz Wasiak, Damian Ciunowicz, Natalia Kapelan, Natalia Szczepaniak, Juliusz Sosnowski, Weronika Goślińska, Jakub Bartkowiak, Agata Budny-Lewandowska, Patrycja Antończyk, Maria Markiewicz, Piotr Gustaw, Kamil Filiks, Maria Jaskólska, Ewelina Stoczyńska-Fidelus

**Affiliations:** 1 Department of Molecular Biology, Chair of Medical Biology, Medical University of Lodz, Zeligowskiego 7/9 St., 90-752 Lodz, Poland; adrianna.rutkowska@umed.lodz.pl (A.R.); tadeusz.strozik@umed.lodz.pl (T.S.); tomasz.wasiak@umed.lodz.pl (T.W.); damian.ciunowicz@umed.lodz.pl (D.C.); natalia.kapelan@umed.lodz.pl (N.K.); natalia.szczepaniak@student.umed.lodz.pl (N.S.); juliusz.sosnowski@stud.umed.lodz.pl (J.S.); weronika.goslinska@student.umed.lodz.pl (W.G.); jakub.bartkowiak@student.umed.lodz.pl (J.B.); agata.budny@student.umed.lodz.pl (A.B.-L.); patrycja.antonczyk@student.umed.lodz.pl (P.A.); maria.markiewicz@student.umed.lodz.pl (M.M.); piotr.gustaw@student.umed.lodz.pl (P.G.); kamil.filiks@student.umed.lodz.pl (K.F.); maria.jaskolska@student.umed.lodz.pl (M.J.); 2 Department of Research and Development, LEK-AM Pharmaceutical Company Ltd., Inwestycyjna 7 St., 95-050 Konstantynow Lodzki, Poland; 3 Student Scientific Circle at the Department of Molecular Biology, Chair of Medical Biology, Medical University of Lodz, Zeligowskiego 7/9 St., 90-752 Lodz, Poland

**Keywords:** CRISPR–Cas9, genome editing, gene therapy, delivery systems, molecular diagnostics, clinical trials

## Abstract

**Highlights:**

**What are the main findings?**

- The review summarizes clinical-stage CRISPR–Cas9 therapies across oncology, monogenic diseases, and immune disorders, highlighting differences between ex vivo edited cell products and in vivo genome editing approaches.
- It outlines the diagnostic framework used in early CRISPR trials, including genotyping for patient selection, quantification of on-target editing, genome-wide off-target detection, and long-term clonal monitoring.

**What are the implications of the main findings?**

- Molecular diagnostics is a critical enabling layer for clinical genome editing, linking editing outcomes with pharmacodynamic effects, safety surveillance, and regulatory evaluation of CRISPR-based ATMPs.
- Standardized sequencing and biomarker strategies will be essential for scaling CRISPR therapies to broader indications while ensuring reliable monitoring of editing outcomes and genome integrity.

**Abstract:**

CRISPR–Cas9 has progressed from an experimental tool to a therapeutic modality, marked by the first regulatory approvals of an ex vivo-edited autologous CD34+ hematopoietic stem cell product that induces fetal hemoglobin (CASGEVY/exa-cel). In this narrative review, we synthesize modality-specific molecular diagnostic strategies used across early CRISPR clinical translation. In parallel, early clinical experience has begun to demonstrate the feasibility of in vivo editing, including subretinal delivery for *CEP290*-associated inherited retinal degeneration (EDIT-101 programme) and hepatocyte-targeted lipid nanoparticles (LNPs) for liver-derived targets such as transthyretin and plasma prekallikrein (KLKB1). As translation expands across hematologic, metabolic, ocular and oncology indications, development is increasingly constrained by the predictability and safety of editing outcomes, delivery-determined biodistribution and exposure time, and immune recognition of bacterial Cas9 orthologs and delivery components. We summarize diagnostic readouts for confirming patient genotype, quantifying on-target editing and expression changes, assessing off-target and structural outcomes using orthogonal assays, and monitoring clonal dynamics and immune responses during long-term follow-up. We also discuss how these readouts interface with CMC controls and regulatory expectations for advanced therapy medicinal products (ATMPs), highlighting the need for fit-for-purpose, standardized testing frameworks in early trials.

## 1. Introduction

**Figure 1:**
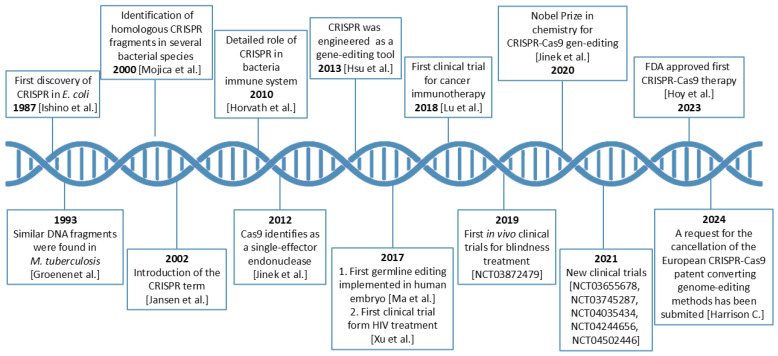
Timeline showing milestone steps in CRISPR–Cas9 development and therapeutic progress, from its identification in *E. coli* in 1987 to the first FDA (U.S. Food and Drug Administration) approval in 2023 for the treatment of sickle cell disease, followed by approval in 2024 for transfusion-dependent β-thalassemia [1,5,25,26,27,28,29,30,31,32,33,34]. Clinical trial identifiers (NCT numbers) correspond to records available in ClinicalTrials.gov. The figure was created by the authors and does not directly reproduce any previously published material. It is based on the general concept presented in Ref. [35], which is distributed under the terms of the Creative Commons Attribution 4.0 International License (CC BY 4.0).

Emmanuelle Charpentier and Jennifer A. Doudna were awarded the 2020 Nobel Prize in Chemistry for developing CRISPR–Cas9 “genetic scissors”, a programmable method for genome editing [1]. Repetitive DNA elements later recognized as CRISPR were first reported in *E. coli* in 1987, and CRISPR arrays were subsequently described across prokaryotic genomes and mechanistically characterized in the 2000s [2,3,4]. The RNA-programmable CRISPR–Cas9 nuclease mechanism was established in 2012 [5]. In its simplest therapeutic configuration, a single-guide RNA directs Cas9 to complementary DNA via RNA–DNA base pairing, introducing a site-specific double-strand break that is repaired mainly by non-homologous end joining or, less frequently, homology-directed repair. Because retargeting requires only a short guide sequence rather than de novo protein engineering, CRISPR–Cas9 is readily scalable and multiplexable compared with earlier platforms such as zinc-finger nucleases and transcription activator-like effector nucleases [6,7]. The resulting ease of design has accelerated translation from basic discovery to clinical-stage genome editing (Figure 1).

In clinical translation, CRISPR–Cas9 now underpins both ex vivo and in vivo somatic gene-editing approaches developed within advanced therapy medicinal product (ATMP) frameworks, including gene therapy medicinal products under EU legislation and European Medicines Agency (EMA) guidance [8,9]. A landmark milestone was the approval of CASGEVY (exagamglogene autotemcel; exa-cel), an autologous CRISPR–Cas9-edited CD34+ hematopoietic stem-cell product: the U.S. Food and Drug Administration approved CASGEVY for sickle cell disease on 8 December 2023, and for transfusion-dependent β-thalassemia on 16 January 2024 [10,11,12]. In this workflow, patient-derived CD34+ cells are collected by apheresis, edited ex vivo by electroporation of a CRISPR–Cas9 ribonucleoprotein complex, cryopreserved, and released against predefined specifications including identity, potency, sterility and molecular characterization of editing outcomes [10,11]. Beyond ex vivo programmes, in vivo CRISPR editing has entered early clinical development; for example, subretinal CRISPR administration targeting *CEP290* in LCA10 demonstrated a favourable safety profile with efficacy signals in a subset of participants in early-phase testing [13].

As the clinical pipeline expands beyond rare monogenic disorders toward broader indications, translational bottlenecks increasingly converge on measurement science. Therapeutic benefit depends on accurate diagnosis and patient stratification (often genotype-defined), robust quantification of on-target editing outcomes (including allele distributions and durability), and sensitive surveillance for rare or delayed risks, including unintended on- and off-target modifications, large structural variants and clonal selection. These needs intersect with evolving expectations for chemistry, manufacturing and controls (CMC), regulatory classification, and long-term follow-up for advanced therapy medicinal products (ATMPs)/gene therapy medicinal products (GTMPs) [8,9,14,15,16,17,18]. In parallel, continued ethical scrutiny, particularly in relation to germline interventions, reinforces the importance of rigorous, transparent evidence generation and post-treatment monitoring for somatic applications [19,20,21,22,23,24].

This narrative review summarizes CRISPR–Cas9 therapeutics with an emphasis on how molecular diagnostics enables patient selection, quantifies on-target editing and edit heterogeneity, supports detection of off-target and structural outcomes, and informs longitudinal monitoring of persistence and clonal dynamics. We further discuss how these readouts integrate with CMC expectations, spanning product characterization and release testing for ex vivo edited cells as well as pharmacodynamic and safety monitoring strategies for in vivo delivery. Together, these considerations link modality-specific constraints to fit-for-purpose endpoints that support interpretation of efficacy and safety in early clinical development.

## 2. CRISPR-Based Gene Editing Platforms for Therapy

Therapeutic genome editing with CRISPR–Cas9 relies on a limited set of mechanisms and delivery strategies that determine which genetic alterations can be achieved safely and efficiently in patients. This section summarizes clinically relevant aspects of CRISPR–Cas9 and outlines major ex vivo and in vivo platforms used or emerging in early-phase gene and cell therapy trials.

### 2.1. Mechanistic Essentials of CRISPR–Cas9

**Figure 2:**
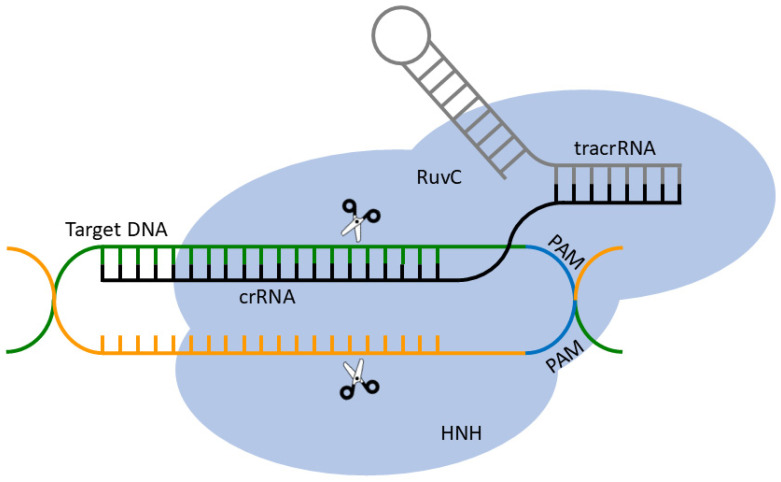
Schematic mechanism of CRISPR–Cas9 immunity and genome editing. In bacteria, CRISPR arrays are transcribed and processed into guide RNAs that, together with Cas9, direct cleavage of complementary target DNA via the HNH and RuvC-like nuclease domains. In therapeutic applications, a synthetic single-guide RNA (sgRNA) performs the same targeting function in engineered cells. Green line: target DNA strand complementary to the crRNA; orange line: non-target DNA strand; black line: crRNA; grey line: tracrRNA. The PAM sequence is indicated on the non-target strand. The R-loop structure formed upon crRNA–DNA hybridization is also shown.

In prokaryotes, CRISPR–Cas systems function as adaptive immune systems in which fragments of foreign nucleic acids (“spacers”) are integrated into CRISPR arrays and later used to guide effector nucleases against matching invaders [25,28,36,37,38,39,40,41,42]. Although multiple classes and types exist [41], therapeutic development has focused almost exclusively on type II systems, where a single multidomain effector protein (Cas9) is directed to a target sequence by a guide RNA. Cas9 contains two catalytic nuclease domains, HNH and RuvC-like, which cleave the complementary and non-complementary DNA strands, respectively, to generate a site-specific double-strand break (DSB) (Figure 2) [5,43,44,45].

In engineered systems, the bacterial CRISPR machinery is simplified to a programmable nuclease complex composed of Cas9 and a single-guide RNA (sgRNA) that fuses the functions of the CRISPR RNA (crRNA) and trans-activating crRNA (tracrRNA) [5,45,46,47]. The sgRNA includes a spacer that base-pairs with the genomic target and a hairpin structure essential for Cas9 binding and activity [47]. Target recognition requires a protospacer-adjacent motif (PAM) immediately downstream of the target sequence; for *Streptococcus pyogenes* Cas9, the canonical PAM is NGG, although engineered variants and alternative Cas proteins recognize broader or distinct PAMs [5,43,48]. PAM binding triggers local DNA unwinding and RNA–DNA pairing, followed by HNH/RuvC-mediated cleavage and formation of a DSB.

Cellular repair of CRISPR-induced DSBs occurs predominantly through non-homologous end joining (NHEJ) or, less frequently, homology-directed repair (HDR). NHEJ operates in dividing and non-dividing cells, is error-prone, and frequently generates small insertions or deletions that disrupt gene function. HDR uses a homologous DNA template to install defined sequence changes, but is largely restricted to S/G2 phases and is strongly influenced by the genomic locus, nuclease format and cell type [49,50]. Donor templates can be supplied exogenously as single-stranded oligodeoxynucleotides, double-stranded plasmids or viral vectors with flanking homology arms [49,50]. The NHEJ and HDR balance, together with nuclease and donor design, determines whether a given therapeutic strategy achieves gene disruption, correction or targeted insertion.

Beyond canonical nuclease-active Cas9, refinements have expanded the therapeutic toolbox. Multiplex editing, where multiple sgRNAs are expressed from the same construct (for example via gRNA–tRNA arrays), enables simultaneous modification of several loci and is increasingly used in T-cell engineering and complex disease models [51]. Catalytically inactive “dead” Cas9 (dCas9), generated by inactivating its nuclease domains, retains programmable DNA binding and can be fused to effector domains to modulate transcription, epigenetic marks or base identity without creating DSBs [52]. These next-generation editors provide additional options for precise (potentially safer) interventions, although most clinical-stage programmes still rely on nuclease-active Cas9 and NHEJ-driven disruption of disease-relevant regulatory elements.

### 2.2. Ex Vivo Genome Editing Platforms

Ex vivo genome editing underpins many CRISPR-based cell and gene therapies in early clinical development. Cells are collected from the patient or a healthy donor, edited in a controlled manufacturing environment, then expanded, tested and administered as a medicinal product. This approach is well suited to hematopoietic stem and progenitor cells (HSPCs) and T lymphocytes, which can be harvested, manipulated and reinfused using established clinical protocols.

In ex vivo workflows, the Cas9–sgRNA complex can be delivered as DNA, RNA or a pre-assembled ribonucleoprotein (RNP). Plasmid or viral vector delivery enables sustained expression but may increase the risk of off-target editing and integration-related events. By contrast, electroporation of Cas9 RNPs provides rapid, transient nuclease activity with a favourable safety profile and is widely used in clinical manufacturing for both HSPC and T-cell products [46]. Process parameters such as cell type, activation state, electroporation conditions and nuclease format substantially affect editing efficiency and cell viability [46,49]. Emerging physical delivery methods, including microfluidic droplet-based mechanoporation, can achieve high editing rates with reduced toxicity and may support more scalable, reproducible manufacturing for future CRISPR-engineered cell therapies [25,46].

Ex vivo CRISPR–Cas9 editing of autologous CD34+ hematopoietic stem and progenitor cells (HSPCs), representing one of the first and most clinically significant applications of ex vivo CRISPR technology, has demonstrated compelling clinical effects in phase 1/2 trials for severe sickle cell disease (SCD) and transfusion-dependent β-thalassemia (TDT). In these studies, mobilized CD34+ cells are collected by leukapheresis, edited in vitro to disrupt the erythroid-specific enhancer of the *BCL11A* gene, and reinfused following myeloablative conditioning to enable bone marrow engraftment. Disruption of *BCL11A* reduces γ-globin repression, reactivating fetal hemoglobin (HbF) and mitigating the pathological effects of defective β-globin. Early clinical data from CLIMB-SCD-121 and related phase 1/2 cohorts demonstrate sustained increases in HbF—average HbF levels exceeded 20% with total hemoglobin > 11 g/dL by three months post-infusion—alongside high editing rates in bone marrow CD34+ cells (≈86%) and durable engraftment. All treated SCD patients remained free of vaso-occlusive crises over approximately 9–32 months of follow-up. Similarly, the majority of TDT patients became transfusion independent, with persistent elevations in HbF over time. Importantly, these benefits have been achieved with a safety profile generally consistent with myeloablative conditioning and autologous transplantation rather than gene-editing-specific toxicity, and without treatment-related deaths or malignancies reported to date. These outcomes support the feasibility of ex vivo CRISPR-HSPC therapy as a potentially curative strategy for hemoglobinopathies [53].

The ex vivo setting enables extensive quality control and molecular characterization of edited cells before administration. Release testing typically includes identity, viability, purity, potency and sterility, plus targeted genotyping of on-target edits and pre-specified off-target sites. Clonal tracking, deep sequencing and functional assays can monitor potential enrichment of unintended variants or transformation-prone clones over time. These capabilities are important for autologous HSPC products with long-term persistence, and for allogeneic T-cell therapies where multiplex editing (for example of *TRAC*, *PDCD1* and *HLA* loci) supports universal “off-the-shelf” products.

### 2.3. In Vivo Delivery Strategies for CRISPR-Based Gene Therapies

In vivo genome editing delivers CRISPR components directly to tissues, avoiding cell collection and reinfusion but requiring efficient, tissue-selective, and well-tolerated delivery with longitudinal safety monitoring. Clinically, the strongest proof-of-concept is liver-targeted LNP delivery in transthyretin (TTR) amyloidosis, first shown in the NTLA-2001 phase 1 report and subsequently extended to broader hereditary ATTR populations and to ATTR cardiomyopathy [54,55,56].

AAV vectors remain attractive for tissues benefiting from durable expression, but their use is constrained by (i) immune responses to capsid/editor and limited redosing, and (ii) tight cargo limits around the wild-type AAV genome size. 

A notable example of in vivo CRISPR–Cas9 delivery is currently being evaluated in the ongoing phase I clinical trial NCT05805007, which investigates the safety and tolerability of ZVS203e, a recombinant adeno-associated virus (AAV)-based CRISPR–Cas9 therapeutic, in patients with retinitis pigmentosa caused by pathogenic *RHO* mutations. In this study, participants receive a single subretinal injection of ZVS203e in one eye, with the CRISPR–Cas9 system designed to selectively disrupt the mutant *RHO* allele, thereby reducing toxic protein accumulation in photoreceptors. The trial uses a dose-escalation design across sequential cohorts to monitor safety, including the incidence of adverse and serious adverse events. Secondary endpoints assess functional retinal outcomes such as best-corrected visual acuity, visual field, retinal thickness, contrast sensitivity, color vision, and multifocal electroretinography, as well as patient-reported vision-related quality of life. This study exemplifies the potential of localized in vivo delivery of CRISPR therapeutics to target compartmentalized tissues, such as the retina, where immune privilege and direct vector administration may maximize efficacy while limiting systemic exposure [57].

Single-vector “all-in-one” designs therefore often rely on compact nucleases and optimized cassettes [58]. Safety concerns include capture/integration of vector-derived DNA at nuclease-induced DSBs, reported at high frequencies in some contexts and capable of perturbing transcriptional architecture at the edited locus [59,60]. A practical workaround for payload complexity is combinatorial vector delivery (separating nuclease and donor template), which has achieved high-efficiency, selection-free knock-in in experimental systems [61].

LNPs provide a modular, non-viral alternative with typically transient editor exposure (mRNA + sgRNA or RNP), narrowing the window for unintended outcomes compared with persistent DNA-based expression [54]. A key advance for RNP–LNP has been thermostable Cas9 engineering (iGeoCas9), enabling robust lung and liver editing after LNP formulation [62]. In parallel, chemistry-driven iteration of ionizable lipids continues rapidly, including biodegradable lipid series reported to improve in vivo performance for nucleic-acid delivery and gene editing [63]. Biomimetic compositions have also been proposed to enhance LNP uptake/trafficking and in vivo editing efficacy in disease models [64].

Beyond AAV and LNPs, multiple preclinical platforms are being developed, including: engineered viral-like particles, polymeric and inorganic nanoparticles, and targeted nonviral systems, to improve cell-type specificity while maintaining transient exposure and scalable manufacturability [65].

Safety assessment is increasingly shifting from small indels and “classical” off-target sites to broader genome-integrity endpoints, including large deletions, rearrangements, and translocations associated with DSB-based editing; these risks can be exacerbated by some HDR-enhancing strategies (e.g., DNA-PKcs inhibition) and motivate dedicated long-read and genome-wide assays [66,67]. Off-target discovery methods are also evolving: DISCOVER-Seq+ was reported to increase sensitivity for in vivo off-target site detection relative to earlier approaches [68]. Finally, mitigation increasingly integrates (i) high-fidelity nuclease variants with guide-dependent efficiency/specificity trade-offs and (ii) uncertainty-aware computational models to improve guide selection [64,69].

Together, the in vivo delivery platform, its persistence, tropism, and immunological profile, defines both tissues and the dominant safety liabilities that must be monitored in CRISPR-based gene therapies [66].

### 2.4. Ex Vivo Versus in Vivo Editing: Implications for Diagnostics, CMC, and Safety Monitoring

The choice between ex vivo and in vivo genome editing determines which critical quality attributes can be measured pre-dose and which risks must be managed post-dose. In ex vivo editing, hematopoietic stem/progenitor cells (HSPCs) or immune effector cells are modified in a controlled manufacturing, enabling pre-administration assessment of identity, editing outcomes, and potency, with molecular assays (e.g., on-target editing distribution, predefined off-target panels, SV-focused assays where relevant) included in release testing as CMC controls. This paradigm underpins late-stage and approved programmes such as exagamglogene autotemcel (exa-cel/CASGEVY) for severe sickle cell disease and transfusion-dependent β-thalassemia [53,70].

By contrast, in vivo editing delivers CRISPR components directly to tissues using vectors such as LNPs or AAV, enabling single-administration strategies but eliminating prescreening of edited cells. Development therefore emphasizes on biodistribution, exposure kinetics, early safety monitoring during peak editor exposure, and pharmacodynamic readouts in circulation or surrogate tissues. First-in-human in vivo CRISPR–Cas9 experience in the liver illustrates this model, including LNP delivery targeting *TTR* (NTLA-2001) and *KLKB1* (NTLA-2002) with pharmacodynamic suppression after a single dose in early-phase studies [54,71].

These differences drive distinct monitoring priorities. For ex vivo HSPC products, follow-up focuses on engraftment durability and lineage output, alongside surveillance for clonal skewing and hematologic transformation, complemented by longitudinal molecular monitoring when indicated [10,72]. For in vivo delivery, early monitoring prioritizes organ-specific toxicity and immunogenicity during the period of maximal editor exposure, supported by fit-for-purpose molecular assays to quantify on-target editing and to assess off-target and structural outcomes when feasible [54,73]. Evidence from LNP-delivered in vivo editing and base-editing studies indicates that tissue tropism, exposure kinetics, and clearance directly shape pharmacodynamic magnitude and early safety signals, effectively dictating sampling plans and diagnostic strategy [74,75].

In parallel, liver-directed base editing programmes targeting *PCSK9* have been supported by robust nonhuman primate data with durable pharmacodynamic effects, illustrating how preclinical PK/PD and safety packages de-risk first-in-human translation when prescreening of edited cells is not possible [75,76].

## 3. Therapeutic Gene and Cell Applications of CRISPR–Cas9

### 3.1. Oncology

Preclinical studies demonstrate that CRISPR–Cas9 can target oncogenic drivers and tumor-supportive pathways in vivo. One approach focuses on chromosomal translocations and fusion oncogenes. In Ewing sarcoma, dual sgRNAs in *EWSR1* and *FLI1* introns disrupt the *EWSR1–FLI1* fusion, ablating the oncogenic allele, suppressing proliferation and slowing tumor growth in mouse models [77]. An analogous strategy has been applied to chronic myeloid leukemia (CML): sgRNAs in *BCR* and *ABL1* introns to create a large deletion in *BCR* and a frameshift in *ABL1*, disrupting *BCR–ABL1*, and reducing proliferation while promoting apoptosis [77]. As *BCR–ABL1* activates JAK/STAT5, RAS/RAF/MEK/ERK and PI3K/AKT/mTOR signaling [78], CRISPR-mediated disruption of the fusion kinase may ultimately be combined with tyrosine kinase inhibitors to deepen responses in CML, although this remains preclinical [77,78].

A second group of interventions targets cell-cycle regulators such as polo-like kinase 1 (PLK1). *PLK1* is overexpressed in many solid tumors and supports a tumor-promoting metabolism and biosynthesis [79,80]. In glioblastoma models, Cas9 mRNA and a *PLK1*-directed sgRNA delivered in targeted lipid nanoparticles induced editing, G2/M arrest and apoptosis. A single intratumoral dose suppressed tumor growth and extended survival in mice, and intraperitoneal administration reduced tumor burden in a metastatic ovarian cancer model [81]. Complementary work using BBB-penetrating nanocarriers achieved robust *PLK1* editing in glioblastoma and extended survival after intravenous dosing [82]. In lung adenocarcinoma, a light-activated nanoparticle platform enables spatial control of *PLK1*-targeted Cas9 editing, limiting off-tumor effect in mice [83].

CRISPR–Cas9 can also be programmed against defined oncogenic point mutations. The EGFR L858R substitution confers constitutive kinase activity in lung adenocarcinoma. In NCI-H1975 cells and xenografts, CRISPR cuts at the L858R locus reduce expression of the mutant allele, impair EGFR signaling and decrease tumor growth [84]. In KRAS-driven non-small-cell lung cancer (NSCLC), synthetic lethality between oncogenic KRAS and CDK4 has been exploited using cell-based carriers delivering CDK4-targeted Cas9, leading to tumor regression in murine models [85,86].

A complementary line of work uses CRISPR to modulate immunologic and death pathways in tumors. In melanoma, transcriptional activation of gasdermin E (GSDME) with dCas9-based activators sensitized tumor cells to cisplatin-induced caspase-3 cleavage, triggering pyroptosis and augmenting antitumor immune responses in primary and recurrent disease models [87,88]. In cervical cancer, a multiplex CRISPR–Cas9 targets *HPV16 E6*/*E7* and the host immune checkpoint PDCD1 (PD-1), increasing apoptosis and enhancing CD8+ T cell activity and inflammatory cytokine production [89,90,91]. In breast cancer, CRISPR-mediated disruption of *IDO1* or the methionine transporter *SLC43A2*, delivered via advanced nanoplatforms, relieves immunosuppression and metabolic competition with T cells, activates STING signaling and restores antitumor immunity in mouse models [92,93,94]. Reprogramming of tumor-associated macrophages (TAMs) is another emerging target: nanovesicles that co-deliver a *PI3Kγ*-targeting Cas9 system and CpG-rich DNA into TAMs shift them from an M2- to an M1-like phenotype and enhance antitumor immune responses [95,96].

Beyond classical oncogenes, CRISPR has been used to probe telomere biology in cancer. The Nage system places Cas9 under the control of an NF-κB-responsive module while directing a telomere-targeting sgRNA to telomeric DNA. Constitutive NF-κB activity in tumor cells drives Cas9 expression and telomere cutting, leading to selective cancer-cell death and tumor growth inhibition in vivo [97]. The Tage system similarly links telomerase activity to Cas9 induction: telomerase extends a single-stranded telomeric substrate on an AAV vector, which recruits a dCas9-VP64/sgRNA complex to activate Cas9 expression, triggering telomere-targeted DNA breaks and apoptosis in telomerase-positive cancers [98]. These switch-like circuits illustrate how CRISPR can be wired to oncogenic signaling or telomere maintenance to restrict editing to tumor cells.

Finally, large-scale CRISPR screens in pancreatic ductal adenocarcinoma (PDAC) and ovarian cancer identify drivers of tumorigenesis, metastasis and drug resistance [99,100,101,102,103,104,105]. For example, in vivo CRISPR libraries in Kras-driven PDAC identify loss of *USP15* or *SCAF1* as accelerators of malignant transformation [99], while genome-wide screens uncover spindle assembly checkpoint genes as central nodes of nab-paclitaxel resistance [101] and define bypass routes that confer resistance to KRAS inhibitors [102]. Although these studies are not therapies per se, they provide a functional roadmap for future genome editing interventions in solid tumors.

From a translational and molecular diagnostics perspective, these studies require sensitive assays to detect editing efficiency and rare events in heterogenous tumors. Amplicon sequencing, ddPCR, and single-cell approaches are increasingly explored to quantify editing outcomes and tumor cell heterogeneity in experimental models.

### 3.2. In Vivo Genome Editing for Monogenic and Metabolic Disorders

The liver is a leading target organ for in vivo CRISPR–Cas9 therapy because of its central role in systemic homeostasis and the natural hepatocyte tropism of lipid nanoparticles (LNPs). The first-in-human NTLA-2001 program for hereditary transthyretin amyloidosis (hATTR) uses LNPs to deliver Cas9 mRNA and a TTR-targeting sgRNA to hepatocytes. A single intravenous dose produced mean serum transthyretin reductions of approximately 50–90% at day 28 in phase 1, with an acceptable short-term safety profile and durable knockdown during follow-up [54,106]. NTLA-2002 applies the same platform to *KLKB1* in hereditary angioedema (HAE), achieving dose-dependent kallikrein suppression and roughly 90–95% reductions in monthly attack rates after a one-time infusion [71,107]. In dyslipidemia, the CTX310 LNP–Cas9 system targets *ANGPTL3*: an ascending-dose phase 1 study reported up to ~70% reductions in circulating ANGPTL3, accompanied by decreases in LDL cholesterol and triglycerides without dose-limiting toxicities [108]. A related program, CTX320, aims to lower lipoprotein(a) by disrupting *LPA* in hepatocytes [109].

Base-editing approaches are now entering the same space. BEAM-302 is an LNP-formulated adenine base editor designed to correct the pathogenic *SERPINA1* variant in alpha-1 antitrypsin deficiency; early clinical data show dose-dependent increases in functional AAT and reductions in mutant Z-AAT after a single infusion [110,111]. Verve Therapeutics is developing *PCSK9*-targeted base editing (VERVE-101/102) to permanently lower LDL cholesterol; interim trials report single-dose LDL-C reductions in the ~40–70% range in high-risk patients [112,113]. AAV-based liver gene transfer of *LIPA* in mice with lysosomal acid lipase deficiency further shows that gene replacement and editing can be combined to correct lysosomal and metabolic phenotypes [114].

Ex vivo editing of autologous hematopoietic stem and progenitor cells (HSPCs) is the most clinically advanced CRISPR modality for hemoglobinopathies. Editing the erythroid enhancer of *BCL11A* derepresses fetal hemoglobin (HbF) and is the mechanism underlying exagamglogene autotemcel (CASGEVY). Across phase 3 trials in severe sickle cell disease (SCD) and transfusion-dependent β-thalassemia (TDT), a single exa-cel infusion after myeloablative conditioning eliminated severe vaso-occlusive crises in the vast majority of SCD patients and produced durable transfusion independence in most TDT patients, with hemoglobin values typically ≥ 10–12 g/dL and high HbF fractions [10,53,70,115]. Alternative strategies edit the *HBG1/HBG2* promoters or repressor binding sites to mimic hereditary persistence of fetal hemoglobin (HPFH), achieving broad F-cell distributions and amelioration of SCD phenotypes [116,117]. High-fidelity Cas9 and AAV6 donor templates can also directly correct pathogenic *HBB* variants in HSPCs at clinically relevant scales, with modeling suggesting that 10–20% corrected stem-cell chimerism may suffice for significant clinical benefit [118,119].

Beyond hematologic disease, CRISPR–Cas9 has been used to correct mutations in cystic fibrosis by editing airway basal stem cells and bronchial epithelial cells, restoring CFTR function in vitro for prevalent variants such as F508del and splice-disrupting alleles [120,121,122,123]. In hemophilia A and B, patient-derived iPSCs carrying intron 1 or intron 22 inversions in *F8* have been corrected via CRISPR with restoration of factor VIII production in differentiated endothelial cells, whereas large-animal models of hemophilia B show that targeted knock-in of human *F9* can alleviate bleeding [124,125,126,127]. In parallel, LNP-mediated CRISPR inactivation of *SERPINC1* (antithrombin) in the liver improves hemostasis in murine HA and HB models, illustrating a “rebalancing” strategy that modulates anticoagulant pathways instead of replacing the missing factor [128].

For inborn errors of metabolism, both in vivo and ex vivo CRISPR strategies have achieved durable benefit in animal models. In hereditary tyrosinemia type I, ex vivo correction of *FAH*-deficient hepatocytes and transplantation lead to stable repopulation of the liver and metabolic rescue [129,130], while in vivo editing can repair the causative *Fah* mutation or inactivate *Hpd* to redirect tyrosine catabolism toward a milder phenotype [129,130,131,132,133]. Phenylketonuria (PKU) has been corrected in mouse models by dual-AAV delivery of Cas9, sgRNAs and repair templates, by FokI–dCas9 nucleases, and more recently by prime and base editors targeting common *PAH* variants, normalizing plasma phenylalanine levels in humanized mice [134,135,136,137]. Glycogen storage disease type Ia, mucopolysaccharidosis type I and related disorders have been ameliorated with gene correction or base editing, where low enzyme restoration (often ~3–1%) is sufficient for phenotypic rescue [138,139,140,141,142,143,144,145,146]. Collectively, these data illustrate that a single-course genome editing intervention can durably correct or modulate diverse monogenic and metabolic conditions.

### 3.3. Neurological, Neuromuscular and Sensory Indications

The eye represents a particularly attractive target for in vivo genome editing not only due to its small, well-defined, and hierarchically organized anatomy but also because of its relatively immune-privileged status. Limited exposure to the immune system reduces the risk of inflammatory responses against viral vectors and CRISPR components, thereby enhancing the safety of genetic interventions. Additionally, the ability to deliver therapy with precision (e.g., via subretinal injection) allows for localized and controlled action while minimizing systemic effects.

The first clinical trial applying CRISPR technology to the retina—EDIT-101—constitutes a breakthrough in the treatment of inherited ocular diseases. This therapy targets a deep-intronic mutation in the *CEP290* gene (variant c.2991+1655A>G), which causes Leber congenital amaurosis type 10 (LCA10). The mechanism of action involves the use of an AAV5 vector to deliver the SpCas9 nuclease and two guide RNAs (gRNAs) that excise the pathogenic fragment responsible for aberrant mRNA splicing [13].

Methodologically, the therapeutic construct was administered via subretinal injection, enabling direct delivery of the vector to photoreceptors and retinal pigment epithelial (RPE) cells, the primary sites of *CEP290* expression. The AAV5 vector was engineered to ensure restricted, local expression of CRISPR components, thereby minimizing the risk of systemic exposure. The use of two gRNAs flanking the mutated region allows precise excision of the aberrant intronic fragment through a double-strand break mechanism, followed by repair via the non-homologous end joining (NHEJ) pathway, ultimately restoring proper transcript splicing.

The phase 1–2 trial was open-label and dose-escalation in design, including cohorts of patients receiving increasing vector doses in a single eye, with simultaneous monitoring of safety and functional vision parameters. Efficacy assessment encompassed a set of prespecified endpoints, including mobility tests under varying lighting conditions (MLMT), full-field stimulus testing (FST), and best-corrected visual acuity (BCVA). Concurrently, adverse events were closely monitored, including inflammatory responses, structural retinal changes assessed by optical coherence tomography (OCT), and potential off-target effects of genome editing.

Results demonstrated that the therapy was well tolerated, with no serious adverse events directly attributable to genome editing, and most patients exhibited improvements in prespecified visual endpoints, including gains in BCVA. These findings highlight the potential of in vivo approaches for treating retinal diseases, particularly in the context of mutations affecting splicing processes, where conventional gene therapy strategies may be insufficient [140,141,142,143].

In Alzheimer’s disease (AD), CRISPR tools have been used to downregulate amyloidogenic genes or modulate the epigenome. A dCas9–DNMT3A fusion targeted to *APP* regulatory regions increased local DNA methylation, reduced APP expression and Aβ production and ameliorated pathology and cognitive deficits in a mouse model [147]. Nuclease-based disruption of the Swedish *APP* mutation (KM670/671NL) in patient-derived fibroblasts and mouse hippocampus lowered Aβ levels [148], while allele-specific editing of PSEN1 mutations decreased the Aβ42/40 ratio in vitro [149,150]. Base-editing approaches that install the protective APP A673T variant or truncate the *APP* C terminus further illustrate how CRISPR derivatives can shift processing away from β-secretase cleavage [151,152]. Allele-selective knockdown of *APOE* ε4 has also been demonstrated using engineered Cas9 variants that preferentially target the ε4 allele in human glia without affecting ε3 [153,154].

For Parkinson’s disease, CRISPR–Cas9 or base editing has been employed to correct or silence key genes in patient-derived iPSCs. Correction of the LRRK2 p.G2019S mutation via HDR or adenine base editing in dopaminergic neurons can normalize disease-related phenotypes, with distinct efficiency–specificity trade-offs [155]. Deletion of *SNCA* in human iPSC-derived dopaminergic neurons reduces their susceptibility to α-synuclein aggregation, supporting a cell-replacement strategy using synucleinopathy-resistant grafts [156]. Broader mitochondrial stress pathways implicated in Parkinson’s disease have been probed by knocking down proteins such as p13, which mitigates toxin-induced dopaminergic neuron loss in mouse models [157].

In spinal muscular atrophy (SMA), base editors and CRISPR nucleases increase exon 7 inclusion in *SMN2*, either by correcting nucleotides in the exon 7 region or disrupting intronic splicing silencers such as ISS-N1. Preclinical data show that such edits can increase SMN protein and ameliorate disease phenotypes, supporting genome editing as a complement to existing antisense therapies [158,159].

CRISPR platforms have progressed in muscular dystrophies and myopathies. In the mdx mouse model of Duchenne muscular dystrophy (DMD), AAV-delivered SaCas9 and sgRNAs flanking exon 23 excise the mutated exon, restore in-frame dystrophin, and improve muscle histology and function [160]. Germline editing in mdx zygotes confirms that sufficient correction prevents the dystrophic phenotype, although such approaches are not clinically acceptable [161]. More recent homology-independent targeted integration (HITI) strategies insert a synthetic “megaexon” 1–19 cassette upstream of intron 19 to reconstitute full-length dystrophin expression from the endogenous locus after Cas9-mediated cutting and NHEJ repair [162]. For Becker muscular dystrophy (BMD), patient-specific iPSC lines have been generated and corrected by inserting missing exons 3–9, restoring dystrophin in derived cardiomyocytes [163], while CRISPR-based deletions of exons 45–47 or 52–55 in mice reproduce BMD-like phenotypes and serve as models for testing genome correction [164,165].

Congenital and metabolic myopathies have been modeled with CRISPR by introducing pathogenic variants into *DNM2*, *ACTA1*, *GAA* and *AGL* in murine or human pluripotent cells, clarifying mechanisms and creating platforms for correction studies [166,167,168,169]. Mitochondrial genome editing remains challenging, but mitochondrially targeted Cas9 constructs indicate that programmable nucleases can access mtDNA and introduce site-specific cuts, even though efficient, precise repair has yet to be demonstrated [170,171].

### 3.4. Viral Diseases and Immune-Related Conditions

CRISPR–Cas9 has reached first-in-human testing against herpes simplex virus type 1 (HSV-1). Building on preclinical work with lentiviral delivery of Cas9 mRNA and dual gRNAs targeting UL8 and UL29 in cornea and trigeminal ganglia [172], a pilot clinical study injected a single dose of a CRISPR formulation into the cornea of three adults with severe refractory herpetic stromal keratitis during keratoplasty. Over roughly 18 months HSV-1 remained undetectable, no off-target edits were found, and no systemic adverse events occurred [173]. These observations support the feasibility and apparent local safety of in vivo CRISPR targeting of DNA viruses in the human eye.

For oncogenic viruses, multiple preclinical strategies have been explored. In Epstein–Barr virus (EBV)-positive malignancies, CRISPR activation with dCas9-VP64 targeted to the *BZLF1* promoter can induce lytic reactivation, sensitizing tumor cells to ganciclovir and selectively eliminating EBV-positive lymphoma and carcinoma cells [174]. In HPV-driven cervical cancer, CRISPR–Cas9 systems that knock out *E6*/*E7* reduce proliferation and increase apoptosis in HPV16-positive cells, and combining this with immune-checkpoint modulation (e.g., PD-1 targeting) further augments antitumor immunity [89,91,175,176].

In HIV-1 infection, the EBT-101 program uses AAV-delivered dual-gRNA CRISPR–Cas9 to excise large fragments of integrated proviral DNA, with an ongoing phase 1/2a trial in aviremic adults on antiretroviral therapy [177,178]. Ex vivo CRISPR ablation of *CCR5* in autologous HSPCs has also been tested in a patient with HIV and acute lymphoblastic leukemia, demonstrating long-term engraftment of edited cells (≈5–8% of marrow cells at 19 months) and a favorable off-target profile, although the editing level was insufficient to allow treatment interruption [31,179]. These efforts illustrate two complementary strategies: direct proviral excision and host-entry blockade.

CRISPR tools are increasingly used to dissect inflammatory and immune pathways relevant to sepsis, chronic lung disease and primary immunodeficiencies. Genome-wide loss-of-function screens in endothelial cells have implicated *NLRX1*, *RIPK3* and *MLKL* in TNF-α cytotoxicity and necroptosis, highlighting nodes that control cell death and vascular injury in sepsis models [180,181]. Knockout of *MUC18* (MCAM/CD146) in primary human airway epithelial cells reduces IL-8 production in response to TLR agonists, identifying MUC18 as a proinflammatory factor in asthma and COPD [182,183]. In JAK3-deficient severe combined immunodeficiency (SCID), CRISPR correction of *JAK3* in patient-specific iPSCs restores T-cell and NK-cell development in vitro, providing a template for gene correction in this and related immunodeficiencies [184]. While these applications remain preclinical, they show how genome editing can chart mechanisms and prototype durable interventions in immune-mediated disorders.

### 3.5. CRISPR-Edited CAR-T Cells and Advanced Cell Therapies

Chimeric antigen receptor (CAR) T-cell therapy is a leading example of living medicinal products, and CRISPR–Cas9 is embedded in the development of allogeneic “off-the-shelf” CAR-T platforms. Conventional autologous CAR-T products rely on patient T cells and viral gene transfer, which can be slow and resource-intensive. Allogeneic approaches use donor T cells edited to remove alloreactive receptors and reduce rejection, enabling batch manufacturing.

Typical edit combinations include: (i) *TRAC* knockout, which eliminates expression of the endogenous *TCR* and lowers the risk of graft-versus-host disease; (ii) *B2M* knockout, which abrogates MHC class I expression and reduces host cytotoxic T-cell recognition; and (iii) *CIITA* knockout, which diminishes MHC class II expression [185,186,187]. Clinical programs such as PACE CART19 (programmable allogeneic CRISPR-edited anti-CD19 CAR-T cells) and CTX110 (allogeneic anti-CD19 CAR-T) use such edits to produce universal CAR-T products for relapsed or refractory B-cell malignancies [188,189]. An overview of registered clinical trials using CRISPR–Cas9-engineered CAR-T cells is provided in Table 1. CTX130, a CD70-targeted CRISPR–Cas9-engineered CAR-T, has shown manageable safety and antitumor activity in T-cell lymphomas in a phase 1 trial [190].

These studies show that multiplex CRISPR editing can be applied at clinical scale to generate standardized allogeneic CAR-T cells. Current challenges include controlling off-target mutations, managing immune recognition of edited cells (including NK-cell responses when MHC class I is absent), and integrating additional edits (e.g., immune checkpoint disruption, safety switches) without compromising cell fitness. Nonetheless, CRISPR-enabled allogeneic CAR-T platforms are among the most advanced examples of CRISPR-derived ATMPs in oncology [19,188,189,190,191,192,193]. To contextualize development beyond CAR-T, Table 2 summarizes registered interventional trials using CRISPR–Cas9 across non–CAR-T indications, including hematologic, metabolic, cardiovascular, ocular, infectious, and immunological diseases.

CRISPR-edited CAR-T therapies also require dedicated molecular monitoring strategies. During manufacturing, targeted sequencing assays are used to verify intended edits and assess predefined off-target sites as part of product characterization. After infusion, longitudinal monitoring focuses on CAR-T cell expansion, persistence, and potential clonal dominance using qPCR/ddPCR, flow cytometry and sequencing-based approaches. These molecular readouts complement clinical endpoints and support long-term safety surveillance for genome-edited cellular therapies.

## 4. Molecular Diagnostics and Trial Readouts in CRISPR-Based Gene and Cell Therapies

The clinical translation of CRISPR-based medicines has accelerated from proof-of-concept studies to late-stage, and in some cases approved, advanced therapy medicinal products (ATMPs). Across ex vivo edited cell products (e.g., autologous edited HSPCs) and in vivo genome editing (e.g., hepatocyte-targeted LNP delivery or locally administered ocular editing), molecular diagnostics serve two functions: (i) patient selection by confirming disease-defining genotypes or molecular phenotypes linked to the mechanism, and (ii) trial readouts to quantify pharmacodynamic (PD) activity, characterize on-target editing outcomes, detect unintended editing (off-target and large structural variants), and support long-term surveillance. These assays are rarely formal “companion diagnostics” (CDx), but function as fit-for-purpose tools enabling clinical decision-making in early-phase trials [8,194,195,196].

### 4.1. Patient Selection and Genotyping

Patient selection spans variant-specific to pathway-based inclusion criteria. Variant-specific enrollment is most evident in monogenic disorders where the hypothesis is anchored to a pathogenic allele or a defined genomic lesion; for example, in vivo retinal editing for *CEP290*-associated inherited degeneration requires baseline confirmation of genotype and molecular defect for eligibility and attribution of benefit [13,197].

By contrast, pathway-based criteria dominate hepatocyte-targeted in vivo editing, where the goal is to modulate a circulating disease-relevant protein rather than correct a patient-unique mutation. In transthyretin amyloidosis, NTLA-2001 (nexiguran ziclumeran) is evaluated with PD anchored to circulating TTR reduction, while eligibility relies on clinical diagnosis (with genotyping differentiating hereditary versus non-hereditary contexts as appropriate) [54,56]. Similarly, NTLA-2002 targets *KLKB1* in hereditary angioedema, where enrollment is defined clinically and by baseline attack burden, supported by kallikrein–kinin pathway biomarkers [71]. In lipid disorders, early-phase *ANGPTL3* editing has relied on baseline lipid phenotyping and target-related biomarkers to contextualize downstream PD effects [114].

Ex vivo edited HSPC therapies for hemoglobinopathies illustrate a mixed paradigm: editing is not individualized to the patient mutation, but inclusion still requires definitive molecular diagnosis (SCD genotype or TDT genotype) plus standardized baseline hematologic assessment. Readouts integrate clinical hematology (hemoglobin, transfusion independence, vaso-occlusive events) with mechanism-linked endpoints such as HbF induction [53,70,198].

Operationally, baseline workflows are typically tiered: (i) orthogonal confirmation of disease-causing variants via targeted sequencing (often NGS panels with confirmatory assays as needed); (ii) documentation of baseline biomarkers aligned to mechanism; and (iii) for cell therapies, immunophenotyping and disease staging to support response attribution and safety interpretation.

### 4.2. On-Target, Off-Target and Clonal Tracking

A central challenge in CRISPR therapeutics is that “editing” is not a single measurable entity, but a distribution of outcomes: precise edits, heterogeneous indel profiles, allele-specific effects, and, in some settings, larger rearrangements (e.g., deletions, inversions, or translocations). Therefore, clinical development relies on a diagnostic stack that characterizes (a) intended on-target modification and (b) unintended genome alterations, with additional layers to monitor clonal dynamics over time [113,199,200,201].

On-target quantification in both ex vivo and in vivo programs commonly uses targeted deep sequencing of the edited locus (amplicon-based NGS) to estimate editing frequency and the spectrum of alleles generated. For ex vivo edited products (HSPCs or CAR-T), this characterization is embedded in manufacturing controls and release testing, and later contextualized in patients through longitudinal hematologic or immunologic outcomes. In hemoglobinopathy programs, HbF induction and distribution of HbF-positive cells (F-cells) are clinically interpretable PD readouts that complement locus-level sequencing, bridging molecular mechanism to patient benefit [53,70].

Off-target assessment is typically performed in a staged manner. Early in development, candidate guides are screened in vitro and/or in relevant primary cells using genome-wide off-target mapping methods that can reveal cleavage at unintended loci. Widely cited platforms include GUIDE-seq (cell-based tag integration at DSB sites), CIRCLE-seq (in vitro circularized DNA substrate), and CHANGE-seq (high-throughput in vitro mapping with scalability and automation advantages) [202,203,204]. These approaches do not replace clinical monitoring but inform risk-based selection of guide RNAs and define a finite list of candidate off-target sites for targeted sequencing in later validation steps. Importantly, CHANGE-seq has highlighted that off-target landscapes are influenced by both sequence context and chromatin-associated features, supporting the need for empirically grounded assay strategies rather than reliance on *in silico* predictions alone [203,204].

Beyond small indels, a clinically relevant diagnostic question is whether nuclease-based editing induces larger structural variants that may be missed by standard amplicon sequencing [200,205]. Long-read sequencing technologies (Oxford Nanopore and PacBio HiFi) have become central to detecting complex structural variation and improving diagnostic yield in settings where short-read approaches underperform, informing expectations for more comprehensive characterization when warranted for genome-editing ATMPs [206]. Amplicon NGS can under-call complex on-target outcomes because large deletions or rearrangements may remove primer-binding sites and introduce amplification bias, distorting allele quantification [199,205]. When warranted, SV-focused assays should therefore be supported by orthogonal confirmation with long-read sequencing (ideally amplification-minimised) to resolve large deletions, inversions and translocations [206]. Computational tools such as Sniffles2, SVIM, cuteSV, and vendor-supported callers (e.g., pbsv) are specifically designed for long-read SV calling and enable efficient detection of variants across different platforms [207,208,209].

Clonal tracking is most critical for therapies involving long-lived, proliferative compartments (notably HSPCs and T cells), because any rare unintended genomic event could expand over time. In ex vivo edited HSPC therapies, clinical follow-up frameworks combine conventional hematology, long-term monitoring for hematologic malignancy signals, and molecular analyses that can detect skewing or emergence of dominant clones [196,210]. For in vivo liver editing programs, where hepatocytes are targeted and the primary PD readouts are circulating proteins, the emphasis shifts toward longitudinal safety monitoring (clinical labs, liver function tests, immunogenicity) coupled with targeted molecular assays where feasible and justified, consistent with long-term follow-up paradigms for gene therapy products [8,196].

In oncology-focused cell therapy programs, including CRISPR-edited CAR-T products, monitoring frameworks integrate cellular kinetics (persistence/expansion), disease response metrics, and immune-related toxicities. Standardized CRS/ICANS grading and cytokine/lab monitoring remain central, while linking these readouts to engineered product design and in vivo behavior [211]. Genome-wide CRISPR screening approaches have continued to identify engineered targets (e.g., RHOG and FAS) that enhance CAR-T potency and durability in preclinical models [212].

### 4.3. Pharmacodynamic and Safety Biomarkers in Early CRISPR Trials

PD biomarkers have been key molecular readouts in early CRISPR trials, providing quantitative evidence of target engagement and pathway modulation. In hepatocyte-directed in vivo editing, PD is often captured by circulating protein knockdown and downstream physiologic effects. In NTLA-2001, serum TTR reduction served as a PD marker of hepatocyte gene disruption, supporting durability and clinical interpretability [54,56]. In NTLA-2002, prekallikrein activity (or related kallikrein–kinin pathway measures) functions as a mechanistically anchored PD endpoint, complemented by clinical outcomes such as attack frequency, an important pairing because it aligns biochemical modulation with patient-relevant benefit [71]. In lipid-focused editing targeting *ANGPTL3*, PD readouts include circulating ANGPTL3 protein and lipid parameters (e.g., triglycerides and LDL-related measures), allowing investigators to link molecular intervention to cardiometabolic risk biomarkers [115].

In ex vivo edited HSPC therapy for sickle cell disease and transfusion-dependent β-thalassemia, PD is frequently framed around HbF biology: HbF levels, total hemoglobin, and the distribution of HbF-positive erythrocytes (F-cells). These markers correlate with fetal hemoglobin reactivation and are measurable over long follow-up [53,70]. Importantly, these PD measures are interpreted alongside transplant-related safety monitoring, engraftment dynamics, and durability of hematologic response, reflecting the fact that the clinical phenotype is shaped by both editing outcomes and autologous transplantation procedures.

Safety biomarkers in early CRISPR trials are multi-layered and reflect both platform risks (editing-associated genomic alterations, immunogenicity to editor components, vector/LNP-related toxicities) and indication-specific risks (e.g., CRS/ICANS in cellular immunotherapy). Across in vivo liver editing programs, safety surveillance has prominently included liver enzymes and broader laboratory chemistry to detect hepatotoxicity or inflammatory responses [54,56]. In cell therapies, cytokine monitoring and standardized toxicity grading remain central, with CRISPR editing adding the requirement to document and mitigate risks related to unintended genomic changes in the engineered cell population [64].

A key differentiator for genome editing ATMPs is the expectation of long-term follow-up for delayed adverse events, including clonal expansion and malignancy signals where biologically plausible. FDA guidance on long-term follow-up after administration of human gene therapy products provides a widely cited framework for the duration and nature of post-treatment monitoring [196]. Within the EU context, EMA guidance on gene therapy medicinal products and investigational ATMP requirements reinforces a risk-based approach to quality characterization and clinical monitoring, supporting the integration of molecular assays as part of a coherent safety strategy rather than isolated exploratory endpoints [8].

Collectively, the emerging diagnostic toolkit for CRISPR therapeutics is converging on three principles. First, fit-for-purpose molecular assays should directly track target engagement through protein and biochemical pharmacodynamic readouts. Second, sequencing-based characterization should robustly capture both intended and unintended editing outcomes, supported by genome-wide mapping approaches during development and by targeted confirmation panels in clinical workflows. Third, longitudinal surveillance should be structured within frameworks that align with regulatory expectations for ATMPs and human gene therapy products. This triad is likely to remain central as CRISPR medicines expand into broader indications, larger patient cohorts, and longer observation periods.

### 4.4. Fit-for-Purpose Assay Validation, CMC Integration and Regulatory-Grade Implementation

In genome editing trials, molecular endpoints must be robust, clinically interpretable, and feasible for longitudinal sampling. Sponsors increasingly justify fit-for-purpose validation aligned with the decision the readout supports (for example, patient selection, dose escalation, lot release, or long-term safety monitoring). In practice, validation typically addresses analytical sensitivity and quantitative range required to resolve low-frequency edits, mosaicism, and rare variants; analytical specificity against homologous loci and common assay artifacts such as PCR recombination, index hopping/barcode cross-talk, and mapping ambiguity in repetitive regions; and commutability and robustness across matrices (whole blood, PBMCs, tissue biopsies, cfDNA) and across time points with variable DNA quality and input.

For on-target editing, short-read amplicon NGS and ddPCR provide scalable quantification of indels and allele fractions, but they can under-represent complex outcomes when large deletions or rearrangements remove primer-binding sites or introduce amplification bias. This limitation is particularly relevant for nuclease-based editing, where double-strand breaks can yield kilobase-scale deletions and more complex local rearrangements [205,213]. When warranted, orthogonal characterization using SV-aware workflows (for example, UDiTaS-style designs for concurrent indel and translocation detection, targeted capture/tiling strategies, UMI-enabled error suppression, and long-read sequencing) increases confidence in the full spectrum of editing outcomes and supports risk-based interpretation for long-lived, clonally expanding cell populations [199].

Off-target assessment similarly benefits from a tiered strategy that combines computational nomination with unbiased or semi-unbiased discovery and targeted confirmation. Genome-wide mapping approaches such as GUIDE-seq and CHANGE-seq have become widely used to define candidate off-target sites for subsequent targeted sequencing, with assay choice informed by modality-specific factors including delivery platform, nuclease exposure window, and tissues plausibly at risk [201,203].

A practical distinction in regulatory-grade implementation is whether editing is performed ex vivo or delivered in vivo. For ex vivo cell therapies, molecular testing bridges manufacturing and clinical follow-up: identity (intended edits and transgene content), purity (residual nuclease/guide, process-related impurities, and defined off-target/SV liabilities where relevant), and potency (mechanism-linked functional readouts) are embedded within a CMC control strategy, supported by chain-of-identity/chain-of-custody and comparability plans for manufacturing changes. For in vivo editing, where the administered “product” is the delivery system and the edited tissue is the biological output, biodistribution, persistence, and clearance are treated as pharmacokinetic-like parameters supported by quantitative molecular assays across relevant compartments. The ICH S12 guideline provides a harmonized framework for nonclinical biodistribution study design and interpretation to support clinical trial planning for gene therapy products [214].

Finally, diagnostics underpins long-term follow-up (LTFU) when delayed adverse events are plausible, including clonal expansion/malignancy signals (particularly in proliferative compartments) or delayed genotoxicity related to unintended on-/off-target outcomes. FDA LTFU guidance and the EMA investigational ATMP guideline support risk-based design across quality, non-clinical, and clinical development. Embedding a pre-specified molecular monitoring plan (sampling schedule, assay hierarchy, confirmatory triggers, interpretation rules) helps connect early PD signals to safety surveillance and downstream endpoints [8,196].

**Figure 3:**
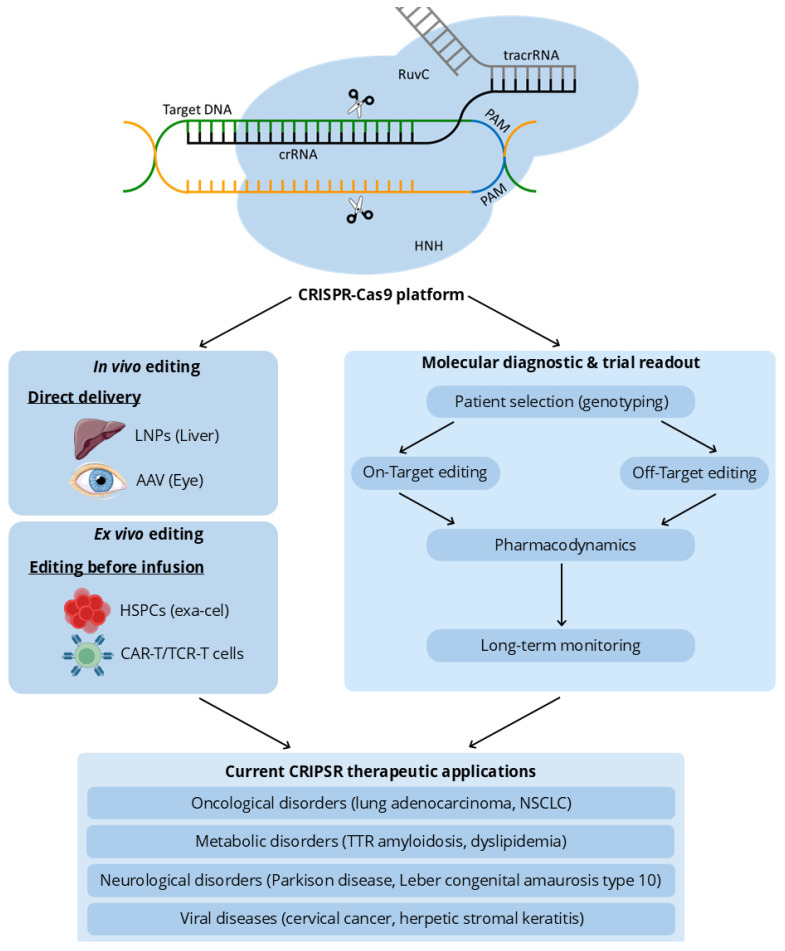
Overview of CRISPR–Cas9 therapeutic strategies and associated molecular diagnostics. CRISPR-based therapies can be broadly divided into ex vivo and in vivo approaches, which differ in delivery, controllability, and safety-monitoring requirements. These strategies are being applied across multiple disease areas, including oncology, monogenic and metabolic disorders, neurological diseases, and infectious conditions. Molecular diagnostics serves as an enabling layer across all modalities, supporting patient selection, quantification of editing outcomes, detection of off-target and structural variants, and long-term safety monitoring.

A summary of the potential applications of CRISPR in both ex vivo and in vivo gene editing, including its use in molecular diagnostics and therapeutic interventions, is presented in Figure 3.

## 5. Challenges and Future Directions with CRISPR–Cas9

CRISPR–Cas9 has moved from discovery to first approval and a widening clinical pipeline. The remaining work centers on three practical areas: safety, delivery, and product design and control. Together, these efforts aim to extend benefits across more tissues and diseases while maintaining a favorable risk profile.

*Safety and genome integrity*. Cas9 creates a double-strand break that normally repairs the intended edit, yet rare imprecise outcomes can occur. Long-read and single-cell studies have shown that on-target repair can generate large deletions and complex structural outcomes in model systems [200,205,213,215]. Accordingly, therapeutic programmes increasingly combine careful guide selection with orthogonal off-target and SV-aware assays (for example, UDiTaS- and CAST-Seq-type approaches) during candidate selection and product characterization to support clinical development [8,199,216]. Early human studies with in vivo and ex vivo CRISPR–Cas9 have reported durable pharmacodynamic effects in the liver and eye, with no treatment-related malignancy signal reported in published clinical datasets to date, although long-term surveillance remains essential [13,53,54,70,71,196].

*Immunogenicity*. A subset of people has preexisting T-cell and antibody reactivity to common Cas9 orthologs, reflecting exposure to the source bacteria [217] Clinical strategies manage this with transient expression, local delivery, and, where appropriate, short immunomodulation. This consideration informs vector choice and dose scheduling rather than blocking development [217].

*Delivery*. Delivery remains a major bottleneck in expanding CRISPR–Cas9 therapeutics beyond tissues accessible to current vector systems, because the delivery platform determines biodistribution, intracellular trafficking, duration of nuclease exposure, repeat-dosing potential, and thus both efficacy and safety [66].

AAV vectors are attractive when durable expression and local delivery are advantageous, as in ocular applications, but their broader use is constrained by limited cargo capacity, anti-capsid and anti-Cas immune responses, restricted redosing, and prolonged nuclease expression; moreover, AAV-derived DNA may be captured at nuclease-induced double-strand breaks, potentially affecting local transcription [13,58,217]. In addition, pre-existing immunity to AAV capsids and Cas proteins further restricts patient eligibility and repeat dosing.

LNPs enable transient systemic delivery and have shown clinically relevant hepatocyte editing, yet they remain strongly liver-directed, while extrahepatic delivery is still limited by poor tissue penetration, inefficient endosomal escape, and dose-related immune activation or hepatic toxicity [54,62,63,71]. However, endosomal escape remains a key inefficiency, with only a small fraction of internalized cargo reaching the cytosol, significantly limiting overall editing efficiency. Newer platforms, including engineered viral-like particles and targeted non-viral systems, may improve transient delivery and cell-type specificity, but they still face challenges related to payload capacity, biodistribution control, and scalable GMP manufacturing [65]. In particular, viral-like particles (VLPs) enable transient, DNA-free delivery of CRISPR components, but their application is currently limited by suboptimal loading efficiency, variable cell-type tropism, and challenges in large-scale production.

Overall, advancing CRISPR therapeutics requires not only improved editors, but also delivery systems that maximize efficiency and tissue specificity while minimizing immunogenicity and toxicity. These limitations highlight the need for delivery systems that combine efficient cellular uptake, controlled biodistribution, and precise cell-type targeting.

*Off-target assessment and control*. Modern pipelines pair computational design with empirical genome-wide profiling. Methods such as GUIDE-seq and CHANGE-seq map cleavage sites and support iterative gRNA optimization, with targeted confirmation used to quantify nominated sites in relevant cellular contexts [193,201].

*Manufacturing and product consistency*. For ex vivo cell products, release testing and comparability frameworks typically integrate molecular characterization (on-target edit distribution; predefined off-target/SV liabilities where relevant) together with potency assays linked to the intended mechanism of action, consistent with ATMP quality expectations [8]. For both ex vivo and in vivo programmes, these controls are complemented by risk-based long-term follow-up when delayed adverse events are biologically plausible [196].

*Ethical and societal context*. Current clinical development is confined to somatic editing. International consensus statements discourage any clinical germline editing [22,24]. For somatic applications, the emphasis is on transparency, robust consent, equitable access, and long-term monitoring [24].

*Active areas to watch*. In vivo LNP-delivered editing continues to expand in liver targets beyond *TTR* and *KLKB1*, including emerging clinical efforts in lipid biology [54,71,108]. In the eye, AAV-delivered ocular editing has advanced to clinical evaluation for *CEP290*-associated disease [13]. Allogeneic, multiplex-edited CAR-T programmes continue to refine edit combinations (including *TRAC*, *B2M*, and *CIITA* disruption strategies) to balance persistence with immune evasion, with additional phase 1/2 readouts accruing [188,189,190].

*Bottom line*. The combination of improved guide design, rigorous genome-wide off-target mapping, SV-aware analytical methods, and fit-for-purpose delivery has moved CRISPR–Cas9 from concept to clinic. Ongoing studies aim to broaden tissue access and durability while continuing to quantify and mitigate low-frequency editing risks.

## 6. Conclusions

CRISPR–Cas systems have moved from proof-of-concept to a credible therapeutic platform, with early trials showing durable benefit when editing, delivery, and patient management are aligned. The key translational bottlenecks now centre less on whether genome editing can work and more on where it can be deployed safely, reproducibly, and at scale within precision medicine development.

For clinical translation, three constraints dominate. First, editing outcomes must be predictable: therapeutic benefit requires not only efficient on-target modification but also control over by-products such as large deletions, rearrangements, and low-frequency off-target events. Second, delivery remains the key determinant of both efficacy and risk, because biodistribution, exposure time, and tissue tropism shape the therapeutic window as well as immunogenicity and toxicity. Third, clinical stewardship must be evidence-driven: trial design should incorporate predefined molecular endpoints that connect editing to pharmacodynamic response and safety signals over time.

In this context, molecular diagnostics is an enabling layer for early clinical CRISPR therapeutics. It supports eligibility and molecular confirmation, quantifies on-target editing and outcome distributions, and underpins surveillance for genotoxicity, clonal dynamics, and immune responses during long-term follow-up. Standardised, sensitive assays, risk-based SV/off-target workflows, and harmonised reporting are essential to compare programmes across modalities and to support regulatory decision-making.

Looking forward, the most impactful advances are likely to come from improving extrahepatic delivery, increasing precision without compromising efficiency, and formalising diagnostic frameworks that bridge research assays to validated clinical readouts. This review outlines how modality-specific constraints map to fit-for-purpose endpoints, CMC control strategies, and long-term monitoring plans that together enable robust interpretation of efficacy and safety in early clinical development.

## Figures and Tables

**Table 1:** Overview of registered clinical trials using CRISPR–Cas9-engineered CAR-T cells for cancer therapy. Gene symbols are in italics. Data extracted from ClinicalTrials.gov (last accessed 8 March 2026).

ClinicalTrials.gov ID	Study Title	Phase	Target Gene	CAR-T-Cell	Disease/Indication
NCT03545815	Study of CRISPR–Cas9 Mediated PD-1 and TCR Gene-knocked Out Mesothelin-directed CAR-T Cells in Patients with Mesothelin Positive Multiple Solid Tumors.	I	*TRAC* and *PDCD1*	Mesothelin- CAR-T	Mesothelin-positive multiple solid tumors
NCT04557436	TT52CAR19 Therapy for B-cell Acute Lymphoblastic Leukemia (B-ALL) (PBLTT52CAR19)	I	*TRAC* and *CD52*	CD19-CAR-T (allogeneic)	Relapsed or refractory B-cell leukemia
NCT04244656	A Safety and Efficacy Study Evaluating CTX120 in Subjects with Relapsed or Refractory Multiple Myeloma	I	*TRAC* and *B2M*	BCMA-CAR-T (allogeneic; CTX120)	Multiple myeloma
NCT04438083	A Safety and Efficacy Study Evaluating CTX130 in Subjects with Relapsed or Refractory Renal Cell Carcinoma (COBALT-RCC)	I	*KO: TRAC, B2M, CD70*	CD70-CAR-T (allogeneic; CTX130)	Relapsed or refractory renal cell carcinoma
NCT06492304	A Safety and Efficacy Study Evaluating CTX131 in Adult Subjects with Relapsed/Refractory Hematologic Malignancies	I/II	*TRAC, B2M, CD70, TGFBR2, ZC3H12A* (Regnase-1)	CD70-CAR-T (allogeneic; CTX131)	Relapsed/refractory hematologic malignancies
NCT05643742	A Safety and Efficacy Study Evaluating CTX112 in Subjects with Relapsed or Refractory B-Cell Malignancies	I/II	*TRAC, B2M, TGFBR2, ZC3H12A (Regnase-1)*	CD19-CAR-T (allogeneic; CTX112)	Relapsed or refractory B-cell malignancies
NCT03747965	Study of PD-1 Gene-knocked Out Mesothelin-directed CAR-T Cells with the Conditioning of PC in Mesothelin Positive Multiple Solid Tumors	I	*PDCD1*	Mesothelin-CAR-T	Mesothelin-positive solid tumors
NCT04035434	A Safety and Efficacy Study Evaluating CTX110 in Subjects with Relapsed or Refractory B-Cell Malignancies (CARBON)	I/II	*TRAC* and *B2M*	CD19-CAR-T(allogeneic; CTX110)	Relapsed/refractory B-cell malignancies
NCT04976218	TGFβR-KO CAR-EGFR T Cells in Previously Treated Advanced EGFR-positive Solid Tumors	I	*TGFBR2*	EGFR-CAR-T	Advanced EGFR-positive solid tumors
NCT05812326	PD-1 Knockout Anti-MUC1 CAR-T Cells in MUC1-Positive Advanced Breast Cancer	I/II	*PDCD1*	MUC1-CAR-T	MUC1-positive advanced breast cancer
NCT04637763	CRISPR-Edited Allogeneic Anti-CD19 CAR-T-Cell Therapy for Relapsed/Refractory B-Cell Non-Hodgkin Lymphoma (ANTLER)	I	*TRAC* and *PDCD1*	CD19-CAR-T (allogeneic; CB-010	Relapsed/refractory B-cell non-Hodgkin lymphoma
NCT04502446	A Safety and Efficacy Study Evaluating CTX130 in Subjects with Relapsed or Refractory T or B-Cell Malignancies (COBALT-LYM)	I	KO: *TRAC*, *B2M*, *CD70*	CD70-CAR-T (allogeneic; CTX130)	T-cell malignancy, Diffuse Large B-Cell Lymphoma
NCT05795595	A Safety and Efficacy Study Evaluating CTX131 in Adult Subjects with Relapsed or Refractory Solid Tumors	I/II	KO: *TRAC*, *B2M*, *CD70*, *TGFBR2*, *ZC3H12A*	CD70-CAR-T (allogeneic; CTX131)	Relapsed/refractory solid tumors
NCT06014073	TRAC and Power3 (SPPL3) Genes Knock-out Allogeneic CD19-targeting CAR-T Cell Therapy in r/r B-NHL	I/II	*TRAC* KO; *SPPL3* (“Power3”) KO	CD19-CAR-T (allogeneic; ATHENA CAR-T)	Relapsed/refractory B-cell non-Hodgkin lymphoma
NCT04767308	A Single-center, Single-arm Exploratory Clinical Trial to Evaluate the Safety and Efficacy of Fully Human Anti-CD5 CAR-T Cells (CT125A) for Relapsed/Refractory CD5+ Hematopoietic Malignancies	I	*CD5* KO (to prevent CAR-T fratricide during manufacturing)	Anti-CD5 CAR-T (CT125A)	Relapsed/refractory CD5+ hematopoietic malignancies
NCT04213469	PD1-CD19-CART in Patients With r/r B-cell Lymphoma	I	PDCD1 locus (site-specific anti-CD19 CAR knock-in with simultaneous *PD-1* disruption)	CD19-CAR-T (autologous; PD1-CD19-CART/BRL-201)	Relapsed/refractory B-cell non-Hodgkin lymphoma
NCT03525782	Anti-MUC1 CAR T Cells and PD-1 Knockout Engineered T Cells for NSCLC	I/II	PDCD1 (edited arm)	anti-MUC1 CAR-T/PD-1-knockout anti-MUC1 CAR-T	Advanced non-small cell lung cancer
NCT03706326	CAR T and PD-1 Knockout Engineered T Cells for Esophageal Cancer	I/II	PDCD1 (edited arm)	anti-MUC1 CAR-T/PD-1-knockout anti-MUC1 CAR-T	Advanced esophageal cancer

Abbreviations: BCMA, B-cell maturation antigen; CAR-T, chimeric antigen receptor T cell; EGFR, epidermal growth factor receptor; KO, knockout; MUC1, mucin 1; NSCLC, non-small-cell lung cancer; PD-1, programmed cell death protein 1; r/r, relapsed/refractory; TCR, T-cell receptor; TGFβR, transforming growth factor beta receptor.

**Table 2:** Overview of registered clinical trials using CRISPR–Cas9-based therapeutics beyond CAR-T. Gene symbols are shown in italics. Data extracted from ClinicalTrials.gov (last accessed 8 March 2026).

ClinicalTrials.gov ID	Conditions/Disease	Phase	Intervention/Treatment	CRISPR–Cas9 Modification
NCT05066165	Acute myeloid leukemia (AML)	I/II	Autologous WT1-directed TCR T cells engineered ex vivo using CRISPR–Cas9	Ex vivo CRISPR–Cas9–engineered TCR-T. Knock-in of WT1-TCR into *TRAC* with knockout of endogenous TCR chains.
NCT03399448	Relapsed/refractory multiple myeloma, melanoma, synovial sarcoma, or myxoid/round cell liposarcoma	I	Autologous NY-ESO-1-directed T cells engineered ex vivo using CRISPR–Cas9	Ex vivo CRISPR–Cas9–engineered TCR-T. Disruption of endogenous TCR and *PDCD1*.
NCT04417764	Advanced hepatocellular carcinoma	I	Procedure: TACE; Biological: PD-1-knockout engineered T cells (autologous)	Ex vivo CRISPR–Cas9 *PDCD1* knockout in T cells.
NCT03044743	EBV-associated malignancies (advanced stage)	I/II	Drug: fludarabine; cyclophosphamide; interleukin-2; Cell therapy: PD-1-knockout EBV-CTLs	Ex vivo CRISPR–Cas9 *PDCD1* knockout in EBV-specific CTLs.
NCT05631912	B-cell non-Hodgkin lymphoma (r/r B-NHL)	I/II	Autologous CD19-STAR-T-cell infusion; Lymphodepletion with fludarabine and cyclophosphamide	*TRAC* KO with knock-in of anti-CD19 STAR construct at *TRAC* (ex vivo CRISPR–Cas9)
NCT06321289			Allogeneic CD19-STAR-T cells	Multiplex CRISPR–Cas9: *TRAC, HLA-A/B*, *CIITA*, *PDCD1* KO; STAR knock-in at *TRAC* via AAV donor (ex vivo)
NCT03728322	β-thalassemia	I	Biological: iHSCs treatment group; Single-center, open-label study of patient-specific induced HSCs	Ex vivo correction of *HBB* in patient-specific iHSCs using CRISPR–Cas9, followed by autologous infusion.
NCT04211480	β-thalassemia	I/II	γ-globin reactivated autologous HSCs	Ex vivo CRISPR–Cas9 editing of autologous HSCs to reactivate γ-globin/HbF via *BCL11A* enhancer disruption.
NCT04205435	β-thalassemia with CVS-654 mutation	I/II	β-globin restored autologous HSCs	Ex vivo CRISPR–Cas9 editing of autologous HSCs for β-globin restoration/correction of the HBB CVS-654 defect.
NCT03655678	Transfusion-dependent β-thalassemia (TDT)	II/III	Biological: CTX001/exa-cel (CASGEVY) – autologous CRISPR-edited CD34+ HSPCs	CRISPR–Cas9 editing of the erythroid-specific enhancer of *BCL11A* in CD34+ HSPCs to upregulate fetal hemoglobin.
NCT04990557	COVID-19 respiratory infection	I/II	Drug: PD-1 and ACE2 knockout T cells	Ex vivo CRISPR–Cas9 knockout of *PDCD1* and *ACE2* in autologous T cells.
NCT04426669	Metastatic gastrointestinal cancers	I/II	Neoantigen-specific TIL with CRISPR-edited checkpoint; lymphodepletion (fludarabine, cyclophosphamide) and IL-2	Ex vivo CRISPR–Cas9 knockout of *CISH* in patient-derived TIL to release intracellular checkpoint restraint.
NCT06783270	Advanced (inoperable) or metastatic melanoma	I	T-cell therapy with CRISPR PD1-edited tumor-infiltrating lymphocytes (TILs)	Ex vivo CRISPR–Cas9–edited TILs with *PDCD1* disruption to silence PD-1.
NCT06379789	Hemophilia B	I/II	REGV131–LNP1265 (in vivo gene insertion therapy)	CRISPR–Cas9–mediated genomic insertion of a functional *F9* (factor IX) transgene in hepatocytes; LNP delivery. Specific nuclease cut site not disclosed in registry.
NCT05120830	Hereditary angioedema	I/II	NTLA-2002 (in vivo CRISPR–Cas9 delivered by LNP)	Knockout of *KLKB1* in liver to durably reduce plasma kallikrein; single-dose LNP formulation.
NCT05144386	HIV-1 infection	I	EBT-101 (AAV9-delivered in vivo CRISPR therapy; single ascending dose)	Multiplex CRISPR–Cas9 excision of integrated HIV-1 proviral DNA using dual guides to remove large genome segments; AAV9 vector.
NCT03057912	HPV-related cervical intraepithelial neoplasia/cervical cancer	I	CRISPR–Cas9 gel or TALEN applied locally; CRISPR–Cas9 plasmid with C32-447 and Poloxamer 407	Local/in situ CRISPR–Cas9 plasmid-based disruption of HPV16/18 *E6/E7* in cervical lesions.
NCT03872479	Leber congenital amaurosis type 10 (*CEP290* IVS26)	I/II	EDIT-101 subretinal AAV5 carrying SaCas9 + dual gRNAs	In vivo CRISPR–Cas9 editing of intronic pathogenic variant in *CEP290* (IVS26) using SaCas9 delivered by AAV5.
NCT05566223	Non–small cell lung cancer (metastatic)	I/II	CISH-inactivated TIL plus lymphodepletion (fludarabine, cyclophosphamide) and IL-2; combination with pembrolizumab per protocol	Ex vivo CRISPR–Cas9 knockout of *CISH* in patient-derived TIL to release intracellular checkpoint restraint.
NCT02793856	Advanced non–small cell lung cancer	I	PD-1 knockout T cells plus cyclophosphamide	Ex vivo CRISPR–Cas9 knockout of *PDCD1* in autologous T cells; first-in-human PD-1–edited T-cell trial.
NCT05662904	Relapsed or refractory acute myeloid leukemia (post–allo-SCT)	I	Donor-derived CD34+ HSC with CRISPR–Cas9-mediated *CD33* deletion plus gemtuzumab ozogamicin	Ex vivo knockout of *CD33* in donor HSC to protect myeloid progeny during anti-CD33 therapy; GO-based conditioning then infusion of edited HSC.
NCT04443907	Sickle cell disease	I	OTQ923 (autologous genome-edited CD34+ HSPCs)	Ex vivo CRISPR–Cas9 editing of autologous CD34+ HSPCs to increase fetal hemoglobin (HbF).
NCT06506461		I	Gene-modified autologous CD34+ cells; conditioning with busulfan; mobilization with plerixafor ± agents per site	Ex vivo CRISPR–Cas9 modification of autologous CD34+ HSPCs for SCD.
NCT04774536		I/II	CRISPR_SCD001 (autologous CD34+ HSPCs)	Ex vivo CRISPR–Cas9 modification of autologous CD34+ HSPCs (“sickle-allele modified”) for severe SCD.
NCT04819841		I/II	Nula-cel (nulabeglogene autogedtemcel)	Precise correction of *HBB* (*HbS*→*HbA*) in autologous CD34+ HSPCs via CRISPR; first-in-human “RESTORE/CEDAR” program.
NCT03745287		II/III	CTX001/exa-cel (autologous CRISPR-edited CD34+ HSPCs)	CRISPR–Cas9 disruption of erythroid *BCL11A* enhancer to derepress *HBG1/HBG2* and induce HbF.
NCT05951205	Sickle cell disease, HbSC genotype	III	Exa-cel (single-dose)	Same mechanism: CRISPR–Cas9 edit at erythroid *BCL11A* enhancer to increase HbF.
NCT05329649	Sickle cell disease (pediatric, HU failure/intolerance)	III	CTX001/exa-cel (autologous CRISPR-edited CD34+ HSPCs)	CRISPR–Cas9 edit of erythroid *BCL11A* enhancer to induce HbF (pediatric cohort).
NCT05477563	Sickle cell disease and transfusion-dependent β-thalassemia	III	CTX001/exa-cel (single-dose autologous CRISPR-edited CD34+ HSPCs)	CRISPR–Cas9 cut in the erythroid *BCL11A* enhancer to derepress *HBG1/HBG2* and induce HbF.
NCT04842812	Advanced solid tumors (engineered TILs/CAR-TILs)	I	Engineered TILs/CAR-TILs targeting HER2, MSLN, PSCA, MUC1, Lewis-Y, GPC3, AXL, EGFR, CLDN18.2/6, ROR1, GD1, B7-H3	Ex vivo CRISPR engineering of TILs; protocol allows inactivation of checkpoint genes such as *PDCD1* and *CTLA4* alongside tumor-antigen targeting.
NCT04925206	Transfusion-dependent β-thalassemia (TDT)	I	ET-01: autologous HSPCs edited ex vivo	Disruption of erythroid *BCL11A* enhancer (+58 DHS) to derepress *HBG1/HBG2* and induce HbF.
NCT05577312		I/II	BRL-101: autologous HSPCs edited ex vivo	Disruption of erythroid *BCL11A* enhancer (+58 DHS) to increase HbF.
NCT05356195	Transfusion-dependent β-thalassemia (pediatric)	III	CTX001/exa-cel: autologous CRISPR-edited CD34+ HSPCs	Editing of erythroid *BCL11A* enhancer to reactivate HbF.
NCT04601051	Transthyretin amyloidosis (ATTR; polyneuropathy and/or cardiomyopathy cohorts)	I	NTLA-2001 (in vivo CRISPR–Cas9, LNP-delivered)	Knockout of *TTR* in hepatocytes to lower serum TTR; Phase 1 study reported durable TTR reductions and acceptable safety.
NCT06128629	ATTR with cardiomyopathy (ATTR-CM)	III	NTLA-2001 vs placebo (MAGNITUDE trial)	Same in vivo CRISPR–Cas9 knockout of *TTR*; Phase 3 efficacy and safety evaluation in ~765 participants.
NCT05210530	Type 1 diabetes mellitus	I	VCTX210A (gene-edited, stem-cell-derived pancreatic endoderm in an implantable device)	CRISPR–Cas9–modified allogeneic pancreatic endoderm cells in a combination product with an implantable delivery device.
NCT05565248		I/II	VCTX211 (gene-edited pancreatic endoderm combination product)	CRISPR–Cas9–modified allogeneic pancreatic endoderm cells in a combination product designed for cell delivery and protection.
NCT07053488	Liver transplantation (prevention of rejection)	I/II	CRISPR-edited HLA donor liver graft to reduce rejection	Ex vivo CRISPR editing of donor liver to knock out HLA class I heavy chains (*HLA-A*, *HLA-B*) and *CIITA* to reduce immunogenicity before transplant.
NCT07170254	HPV-16–related cervical HSIL	II	BD114: lentiviral-like particles delivering CRISPR for local treatment of HPV16 HSIL	In vivo CRISPR–Cas payload designed to disrupt HPV16 *E6*/*E7* in cervical HSIL lesions.
NCT05805007	Retinitis pigmentosa caused by *RHO* mutation	I/II	ZVS203e, single subretinal dose of rAAV-CRISPR	In vivo CRISPR–Cas9 editing to silence/disrupt mutant *RHO* in photoreceptors after subretinal delivery.
NCT06465537	Primary open-angle glaucoma with *MYOC* mutation	I	BD113 (BD113vLVP), CRISPR–Cas9 instantaneous gene-editing therapy	In vivo CRISPR–Cas9 editing targeting mutant *MYOC* to reduce intraocular hypertension in POAG.
NCT04560790	Refractory herpetic viral keratitis	I	BD111 CRISPR–Cas9 mRNA instantaneous gene-editing therapy	Local/in situ CRISPR–Cas9 therapy delivered by corneal injection to cleave the HSV-1 genome in herpetic keratitis.
NCT06474442	Herpes simplex virus type I stromal keratitis	II	BD111 injection plus standard therapy vs. standard therapy	Local/in situ CRISPR–Cas9 therapy targeting the HSV-1 genome in stromal keratitis.

Abbreviations: AML, acute myeloid leukemia; ATTR, transthyretin amyloidosis; ATTR-CM, transthyretin amyloidosis with cardiomyopathy; B-NHL, B-cell non-Hodgkin lymphoma; EBV, Epstein–Barr virus; EBV-CTL, EBV-specific cytotoxic T lymphocyte; HbA, adult hemoglobin; HbF, fetal hemoglobin; HbS, sickle hemoglobin; HSIL, high-grade squamous intraepithelial lesion; HSC, hematopoietic stem cell; HSPC, hematopoietic stem and progenitor cell; LNP, lipid nanoparticle; r/r, relapsed/refractory; SCD, sickle cell disease; STAR-T, synthetic T-cell antigen receptor T cell; TACE, transarterial chemoembolization; TCR-T, T-cell receptor-engineered T cell; TDT, transfusion-dependent β-thalassemia; TIL, tumor-infiltrating lymphocyte; WT1, Wilms tumor 1.

## Data Availability

No new data were created or analyzed in this study.

## Abbreviations

The following abbreviations are used in this manuscript:

AAT	alpha-1 antitrypsin
Aβ	amyloid-β peptide
AAV	adeno-associated virus
AAV5	adeno-associated virus serotype 5
AAV6	adeno-associated virus serotype 6
ABCA4	ATP-binding cassette subfamily A member 4 (gene)
ABL1	ABL proto-oncogene 1 (gene)
ACTA1	actin alpha 1, skeletal muscle (gene)
AD	Alzheimer’s disease
AKT	AKT serine/threonine kinase (protein kinase B)
ANGPTL3	angiopoietin-like 3 (gene/protein)
APOE	apolipoprotein E (gene/protein)
APP	amyloid precursor protein (gene/protein)
ATMP	advanced therapy medicinal product
B2M	beta-2 microglobulin (gene/protein)
BCL11A	B-cell lymphoma/leukemia 11A (gene)
BCR	breakpoint cluster region (gene)
BEAM-302	clinical programme code (base editing)
BMD	Becker muscular dystrophy
BZLF1	EBV immediate-early gene *BZLF1*
CAR	chimeric antigen receptor
Cas9	CRISPR-associated protein 9
CASGEVY	exagamglogene autotemcel (exa-cel)
CAST-Seq	sequencing assay for detection/characterization of chromosomal rearrangements/translocations
CD19	cluster of differentiation 19
CD34+	CD34-positive (hematopoietic progenitor marker)
CD70	cluster of differentiation 70
CDK4	cyclin-dependent kinase 4
CEP290	centrosomal protein 290 (gene)
cfDNA	cell-free DNA
CHANGE-seq	genome-wide off-target mapping method
CIITA	class II major histocompatibility complex transactivator (gene)
CIRCLE-seq	in vitro genome-wide off-target mapping method
CMC	chemistry, manufacturing and controls
CML	chronic myeloid leukemia
CpG	cytosine–phosphate–guanine (CpG motif/oligodeoxynucleotides)
CRISPR	clustered regularly interspaced short palindromic repeats
CRS	cytokine release syndrome
CTX110	clinical programme code (allogeneic anti-CD19 CAR-T)
CTX130	clinical programme code (CD70 CAR-T)
CTX310	clinical programme code (in vivo editing)
CTX320	clinical programme code (in vivo editing)
dCas9	catalytically inactive (“dead”) Cas9
DMD	Duchenne muscular dystrophy
DNM2	dynamin 2 (gene)
DNMT3A	DNA methyltransferase 3A
DNA-PKcs	DNA-dependent protein kinase catalytic subunit
DSB	double-strand break
EBT-101	clinical programme code (HIV-1 CRISPR)
EDIT-101	clinical programme code (ocular in vivo CRISPR)
ERK	extracellular signal-regulated kinase
EWSR1	EWS RNA binding protein 1 (gene)
exa-cel	exagamglogene autotemcel
F8	coagulation factor VIII (gene)
F9	coagulation factor IX (gene)
FAH	fumarylacetoacetate hydrolase (gene/protein)
FAS	Fas cell surface death receptor (gene/protein)
FDA	U.S. Food and Drug Administration
FLI1	FLI1 transcription factor (gene)
GAA	acid alpha-glucosidase (gene)
GSDME	gasdermin E (gene/protein)
GTMP	gene therapy medicinal product
GUIDE-seq	genome-wide off-target mapping assay (tag-integration based)
HA	hemophilia A
HAE	hereditary angioedema
HBB	hemoglobin subunit beta (gene)
HBG1	hemoglobin subunit gamma 1 (gene)
HBG2	hemoglobin subunit gamma 2 (gene)
HDR	homology-directed repair
hiPSC	human induced pluripotent stem cell
HITI	homology-independent targeted integration
HLA	human leukocyte antigen
HNH	HNH nuclease domain (Cas9)
HPFH	hereditary persistence of fetal hemoglobin
HPD/Hpd	4-hydroxyphenylpyruvate dioxygenase (gene)
HSV-1	herpes simplex virus type 1
hATTR	hereditary transthyretin amyloidosis
ICANS	immune effector cell-associated neurotoxicity syndrome
ICH	International Council for Harmonisation
IDO1	indoleamine 2,3-dioxygenase 1 (gene/protein)
iGeoCas9	thermostable engineered Cas9 variant (as named in text)
iPSC	induced pluripotent stem cell
ISS-N1	intronic splicing silencer N1
JAK3	Janus kinase 3
KLKB1	kallikrein B1 (plasma kallikrein; gene)
KRAS	KRAS proto-oncogene (gene)
LCA10	Leber congenital amaurosis type 10
LDL	low-density lipoprotein
LDL-C	low-density lipoprotein cholesterol
LRRK2	leucine-rich repeat kinase 2 (gene/protein)
LTFU	long-term follow-up
MCAM	melanoma cell adhesion molecule (CD146)
MEK	mitogen-activated protein kinase kinase
MHC	major histocompatibility complex
MLKL	mixed lineage kinase domain-like pseudokinase
mTOR	mechanistic target of rapamycin
MUC18	MUC18/MCAM/CD146
Nage	NF-κB-activated gene editing (system name)
NF-κB	nuclear factor kappa-B
NGG	canonical PAM motif for SpCas9
NHEJ	non-homologous end joining
NK	natural killer (cells)
NTLA-2001	clinical programme code (*TTR* editing)
NTLA-2002	clinical programme code (*KLKB1* editing)
PAH	phenylalanine hydroxylase (gene)
PAM	protospacer-adjacent motif
PACE CART19	programmable allogeneic CRISPR-edited anti-CD19 CAR-T (programme name)
PBMC	peripheral blood mononuclear cells
PCSK9	proprotein convertase subtilisin/kexin type 9 (gene/protein)
PDAC	pancreatic ductal adenocarcinoma
PDCD1	programmed cell death 1 (gene; PD-1)
PI3K	phosphoinositide 3-kinase
PI3Kγ	phosphoinositide 3-kinase gamma
PK/PD	pharmacokinetic/pharmacodynamic
PLK1	polo-like kinase 1 (gene/protein)
PSEN1	presenilin 1 (gene/protein)
RAF	RAF kinase (pathway node)
RAS	RAS GTPase (pathway node)
RNP	ribonucleoprotein
RIPK3	receptor-interacting serine/threonine-protein kinase 3
RuvC	RuvC-like nuclease domain (Cas9)
SaCas9	*Staphylococcus aureus* Cas9
SCAF1	SR-related CTD-associated factor 1 (gene)
SCID	severe combined immunodeficiency
SERPINA1	serpin family A member 1 (gene)
SERPINC1	serpin family C member 1 (antithrombin; gene)
sgRNA	single-guide RNA
SLC43A2	solute carrier family 43 member 2 (gene)
SMA	spinal muscular atrophy
SMN2	survival of motor neuron 2 (gene)
SNCA	synuclein alpha (gene)
SpCas9	*Streptococcus pyogenes* Cas9
STING	stimulator of interferon genes
SV	structural variant
Tage	telomerase-activated gene editing (system name)
TAM	tumor-associated macrophage
TLR	Toll-like receptor
TNF-α	tumor necrosis factor alpha
TTR	transthyretin
UDiTaS	Uni-Directional Targeted Sequencing
UMI	unique molecular identifier
USP15	ubiquitin specific peptidase 15 (gene)
VP64	VP16-derived transcriptional activation domain (tetramer; used in dCas9-VP64)
Z-AAT	Z variant of alpha-1 antitrypsin
